# Proximal humeral fractures in the elderly. Treatment with an intramedullary nail.

**DOI:** 10.1007/s00068-025-02926-3

**Published:** 2025-08-11

**Authors:** T. Sigterman, J. Verbruggen

**Affiliations:** Department of Trauma Surgery, Maastricht University Medical Center, P.Debeyelaan 25, P.O. Box 5800 6202 AZ, Maastricht, The Netherlands

**Keywords:** Fracture, Proximal humerus, Osteosynthesis, Intramedullary nail

## Abstract

**Purpose:**

To discuss the use of intramedullary nailing in the treatment of proximal humeral fractures in the elderly.

**Method:**

Review of the existing literature on intramedullary nailing of proximal humeral fractures in the elderly.

**Results:**

Intramedullary nailing of proximal humeral fractures as minimal invasive technique has certain advantages in view of soft tissue damage, blood loss and operation time. On the other hand it might not be the ideal solution for more complex fracture types.

**Conclusion:**

Intramedullary nailing of proximal humeral fractures in the elderly is a valid treatment option with some advantages compared to plating. However operative treatment of these fractures still has a large re-operation rate compared to non-operative treatment.

## Introduction

The proximal humeral fracture is still debated as the unsolved fracture. Especially proximal humeral fractures in the elderly remain a challenge. Different treatment options exist, from non-operative over osteosynthesis with nail or plate to prosthetic replacement. The optimal technique is still debated in the literature. In this paper we will describe and discuss the use of intramedullary nailing for proximal humeral fractures in the elderly illustrated by 2 cases.

## Technique

As with the use of locking plates, achieving an anatomical reduction is crucial prior to inserting the intramedullary nail. This includes restoring the correct head-shaft angle and restoring the medial hinge to prevent varus displacement [1, 2]. The use of an intramedullary nail however offers several advantages in the treatment of elderly patients with osteoporotic proximal humeral fractures. This minimally invasive technique results in reduced soft tissue damage and shorter operative times, which may, in turn, minimize perioperative and postoperative complications [3].

The intramedullary nail functions as a load-sharing device, meaning that stability is not reliant on the fixation of screws in osteoporotic cancellous bone. Instead, the nail itself provides the necessary reduction and stability of the humeral head fragment, while the locking screws ensure axial and rotational stability. Biomechanically, the intramedullary nail has been shown to be superior to locking plates [4–6].

Humeral nailing may be indicated in cases involving pathologic fractures, surgical neck fractures, or combined fractures of the proximal humerus and humeral shaft. However, there is still no clear consensus regarding fracture types that definitively favor intramedullary nailing over plate fixation. Some studies suggest that for complex fractures, particularly three- and four-part fractures, or for fractures with significant displacement (> 5 mm) of the tuberosities, intramedullary nailing may not be the best treatment option [7, 8]. Intramedullary locking nails were used for two-part fractures or three- and four-part fractures with metaphyseal or diaphyseal involvement and no significant displacement of the tuberosities [9] Over the past decade however several studies have reported favorable outcomes for 3- and 4-part fractures with low complication and nonunion rates, good patient reported outcomes, and positive functional results such as range of motion and strength. [10–12] Overall, with improved implant design for intramedullary devices combined with biomechanical benefits, good results in more complex fracture have been published.

Earlier nails like the Polaris led to good healing and functional results, but loosening of screws with secondary dislocation of the nail and hence the fracture were main complications [2, 13]. After nails with angular stable locking bolts had been introduced, results became better [1].

Various designs of intramedullary nails are available nowadays, including straight and curved types. The general idea is that curved nails should be introduced just lateral to the humeral head fragment. However, this can cause dislocation of the head fragment or fracture of the greater tuberosity. In cases of metaphyseal comminution, there is a risk of varus displacement [14]. Postoperative pain and irritation of the rotator cuff, which can compromise shoulder function, are potential complications [15]. Therefore, it is recommended to insert both curved and straight nails through the head fragment. The nail should be advanced into the subchondral bone to prevent protrusion into the subacromial space while simultaneously anchoring in the subchondral bone plate of the humeral head, stabilizing the head fragment and preventing varus displacement. Locking screws provide both rotational and axial stability. Similar to plate fixation, locking screws must be well advanced into the subchondral bone for optimal purchase. Additional screws or sutures may be used to reduce and secure the tuberosities, counteracting the varus forces exerted by the rotator cuff [16]. A nail offers the necessary support for the humeral head at the medial cortex in cases of medial comminution. Depending on the nail design, locking screws can be placed as medial support for the head fragment [6].

**Fig. 1:**
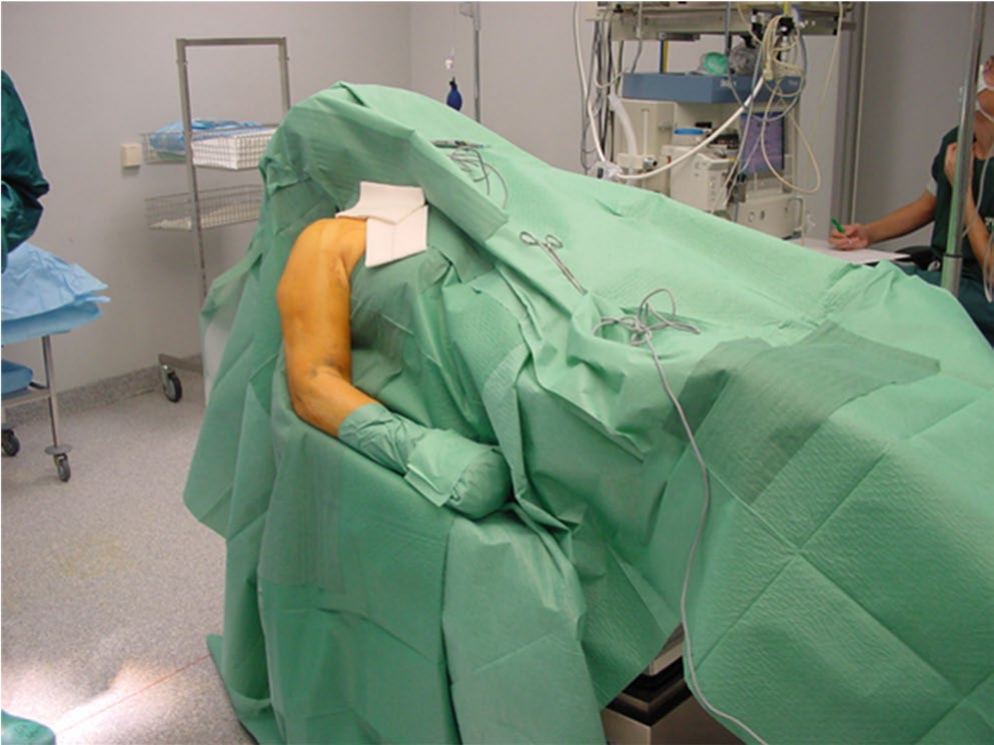
Beach chair position of the patient

**Fig. 2:**
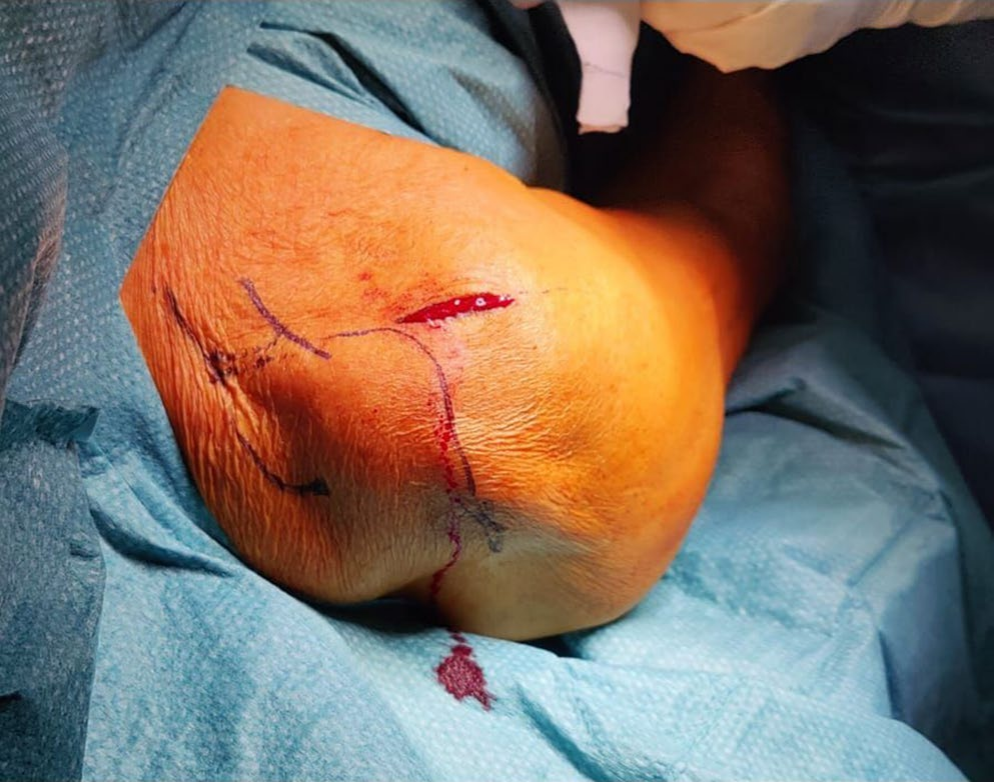
Anterolateral incision over the humeral head

**Fig. 3:**
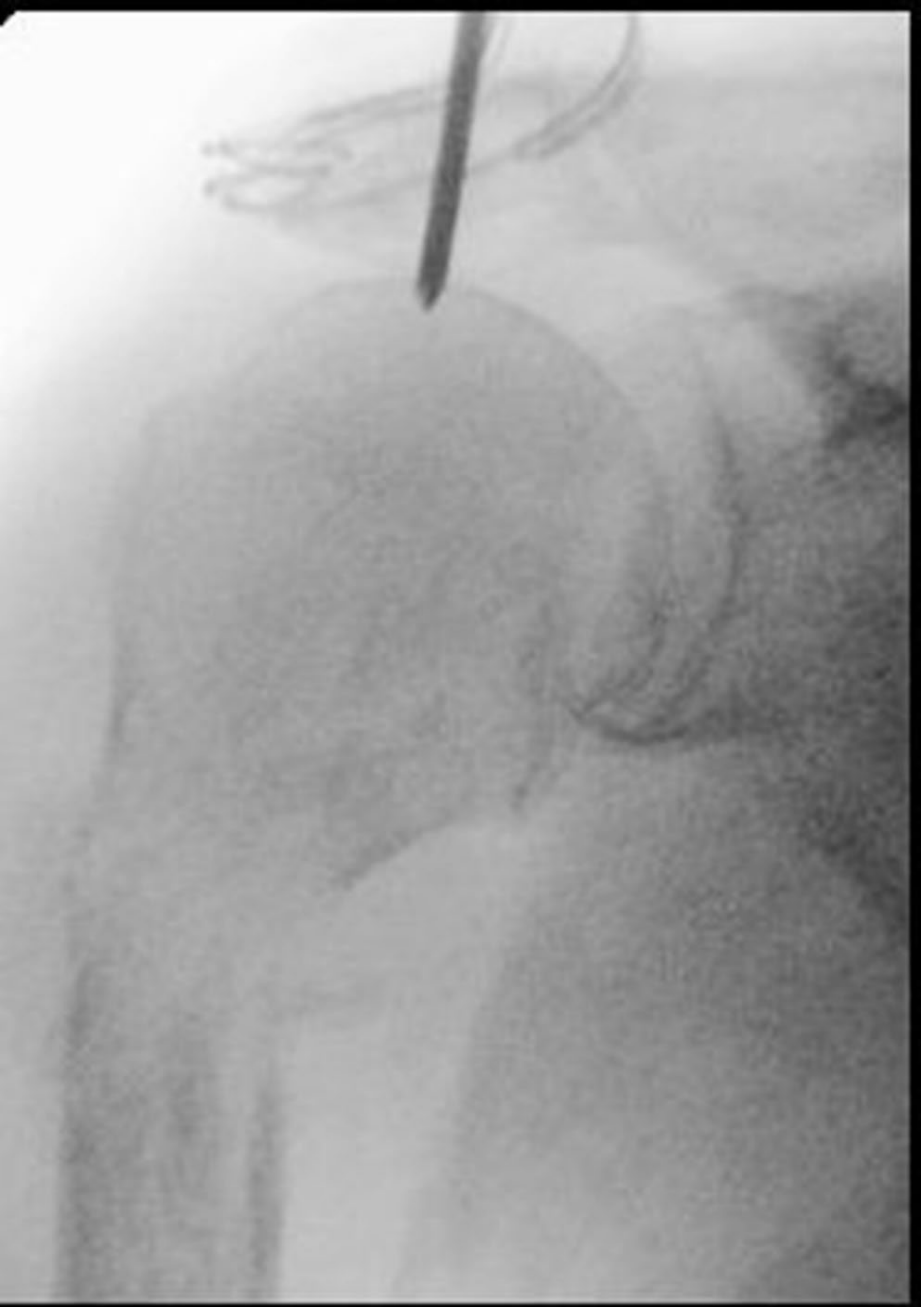
Point of introduction for the nail

**Fig. 4:**
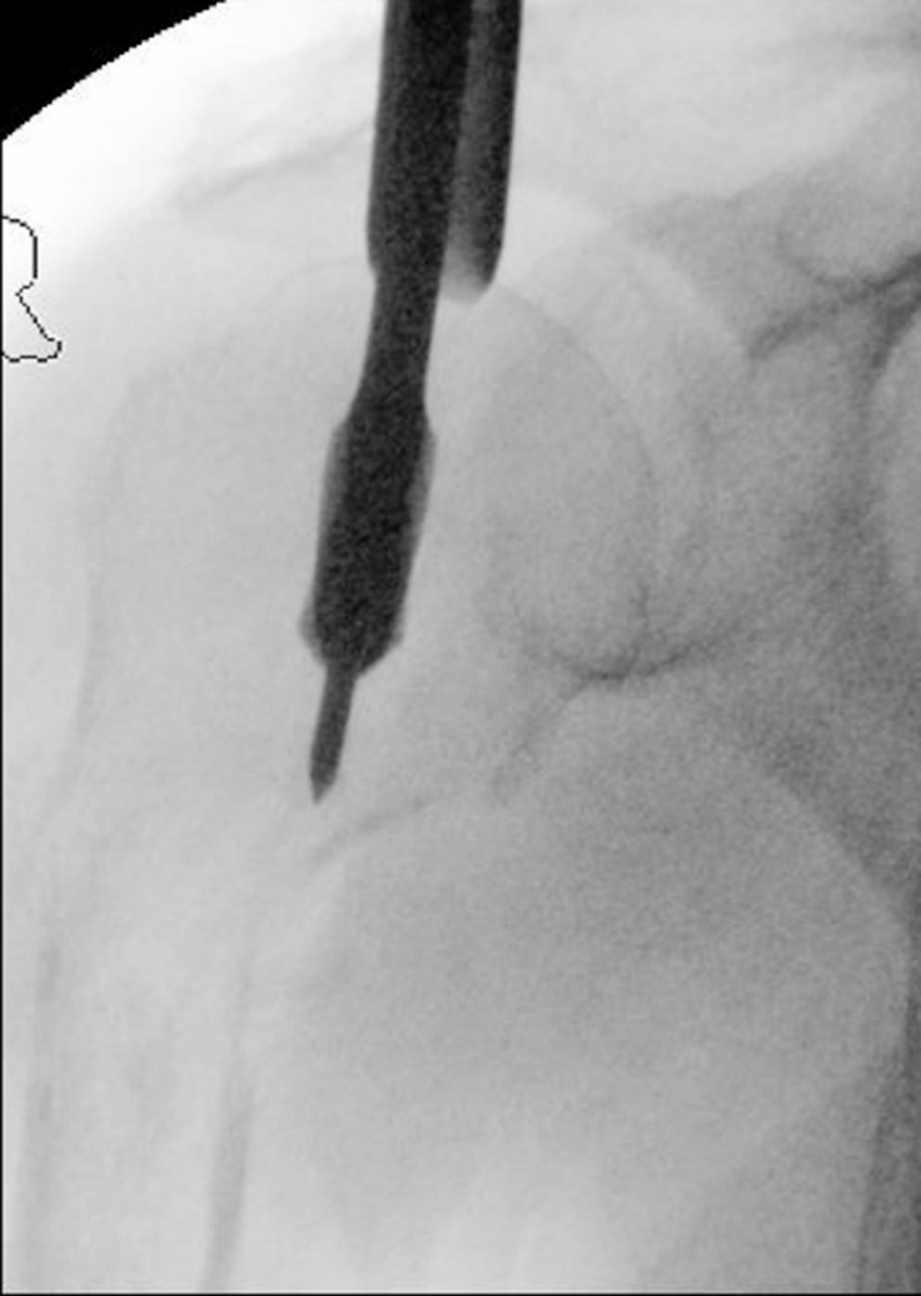
Creating access through the humeral head with the burr

**Fig. 5:**
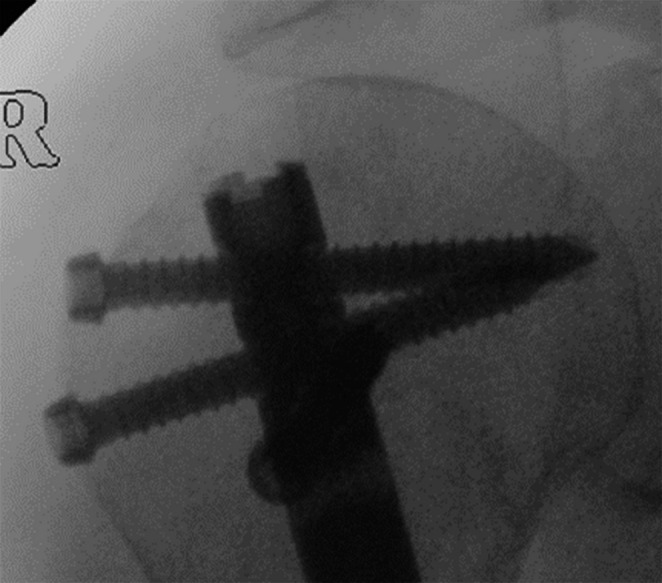
End result with properly countersunk nail

In general proximal humeral nails are best introduced with the patient in beach chair position, the affected arm supported by an arm rest (Fig. 1). After disinfection and sterile draping a small deltoid split incision is made anterolateral from the acromion (Fig. 2), the deltoid and supraspinate muscles are split along the fibers and the medullary canal is opened medial from the footprint of the supraspinate muscle through the humeral head fragment in line with the humeral shaft (Figs. 3 and 4). After preparing the medullary canal, the nail can be inserted. The nail is advanced in the humeral head making sure it is completely countersunk and anchored in the subchondral bone stabilizing the head fragment (Fig. 5). Postoperatively early mobilization of the shoulder is allowed.

We present two illustrative cases of proximal humeral nailing.

### Case 1 Ms. G., 67y

This 67 year old lady was hit at low speed by a car while riding her bike and fell on her left shoulder. Her medical history mentioned osteoporosis for which she has been treated with bisphosphonates.

**Fig. 6:**
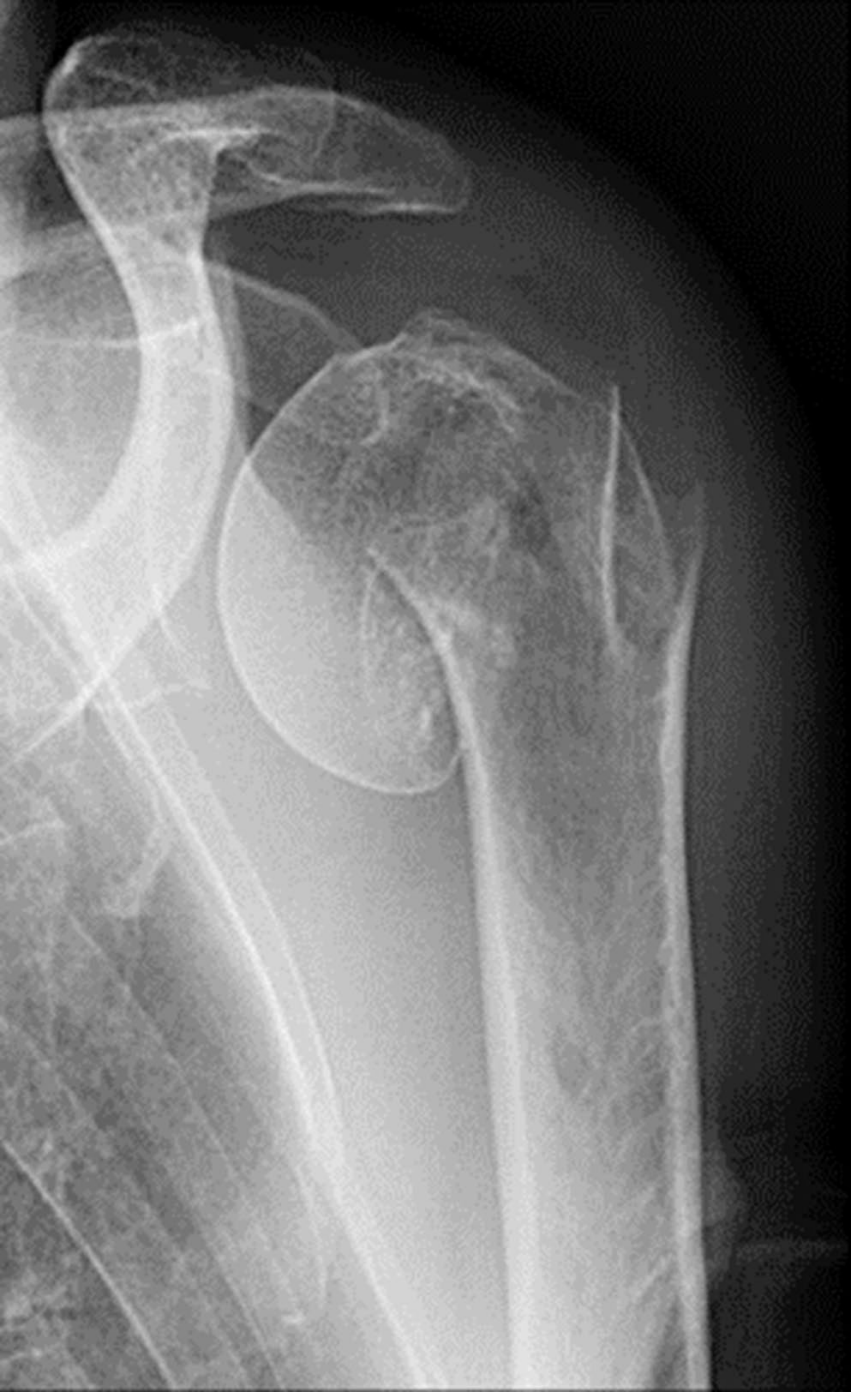
Varus displaced fracture

**Fig. 7:**
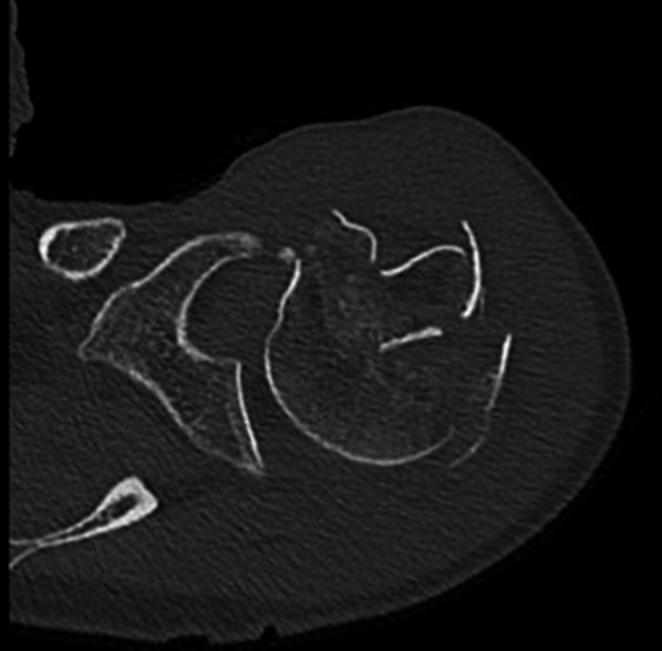
CAT-scan showing the 4-part fracture

She was admitted to our emergency department. The routine trauma screening revealed a displaced 3-part proximal humeral fracture on the left side (Fig. 6). There were no other lesions. A CAT-scan showed a 4-part proximal humeral fracture according to the Hertel criteria (Fig. 7).

The fracture was treated with an intramedullary nail (T_2_ Stryker®). The postoperative images showed a good reduction of the fracture with a good position of the intramedullary nail. The postoperative course was uneventful. During follow up she was regularly seen in our out patient’s department. She showed a good recovery of shoulder function and a gradually healing of the fracture.

**Fig. 8:**
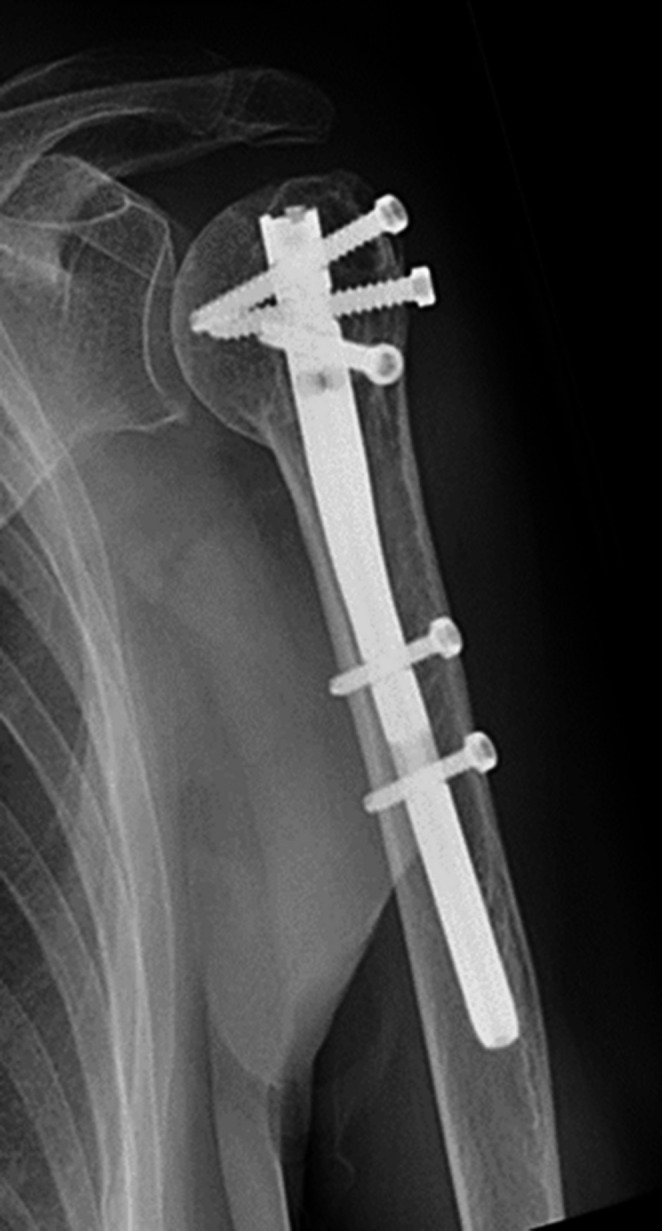
Healed fracture. AP-view

**Fig. 9:**
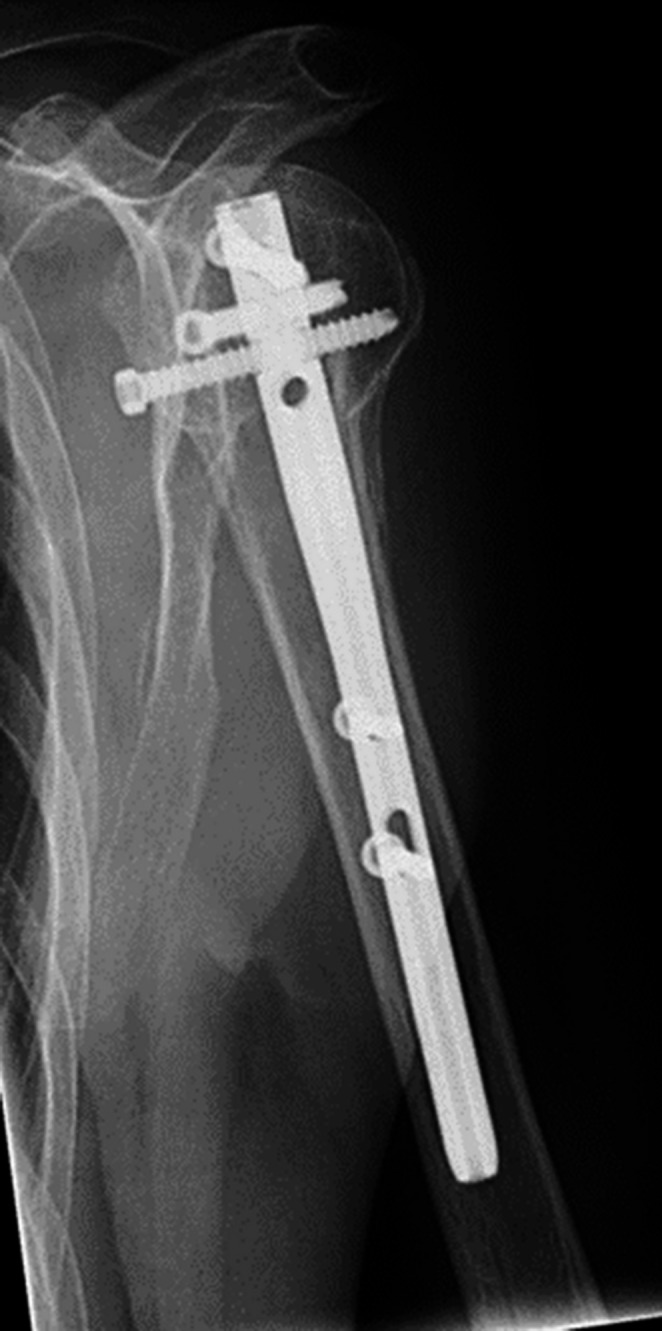
Lateral view after healing

One year postoperatively the fracture was completely healed (Figs. 8 and 9). The patient had regained a good shoulder function with an abduction of 120° and an forward flexion of 150°. The Constant & Murley score after one year was 62 points on the left side which is comparable with the 66 points for the contralateral side. She was released from follow up.

## Case 2 Ms. M.,72 y

This 72 years old female patient fell from standing height on both of her arms.

She was admitted to the ER with complaints of pain over the left shoulder and hemi-thorax.

**Fig. 10:**
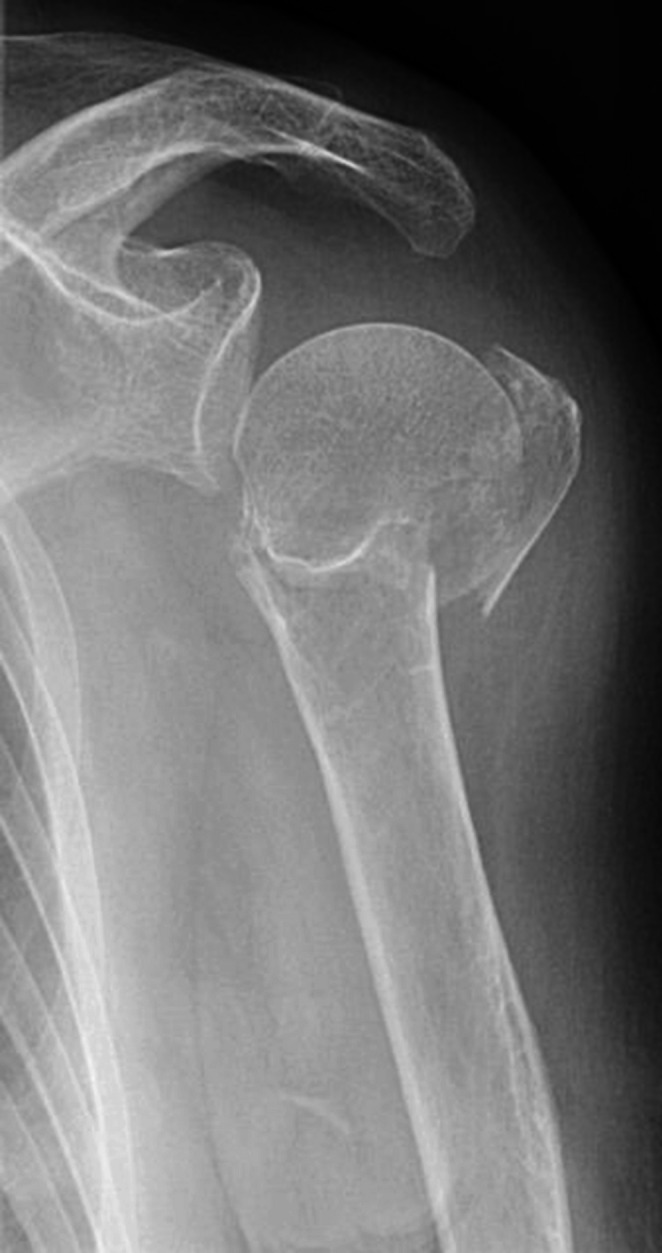
Valgus displaced proximal humeral fracture

A routine trauma screening revealed a proximal humeral fracture on the left side (Fig. 10).

**Fig. 11:**
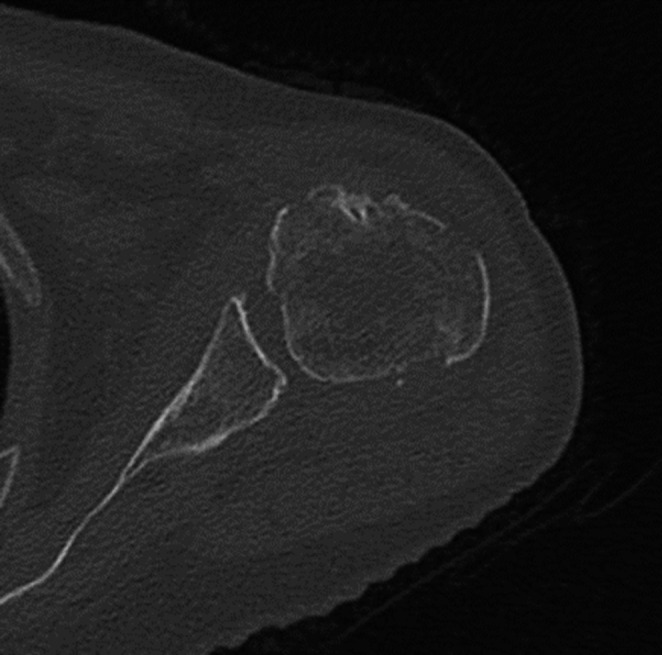
CAT-scan of the shoulder showing the 4-part fracture

No other fractures or lesions were diagnosed. A CAT-scan of the shoulder showed a 4-part proximal humeral fracture according to the Hertel classification (Fig. 11).

A conservative treatment was started but after a week a control radiography showed a further displacement of the head fragment indicating instability.

She underwent an osteosynthesis with an intramedullary nail (T_2_ Stryker®). Postoperative radiographies showed a good fracture alignment with a good position of the nail.

**Fig. 12:**
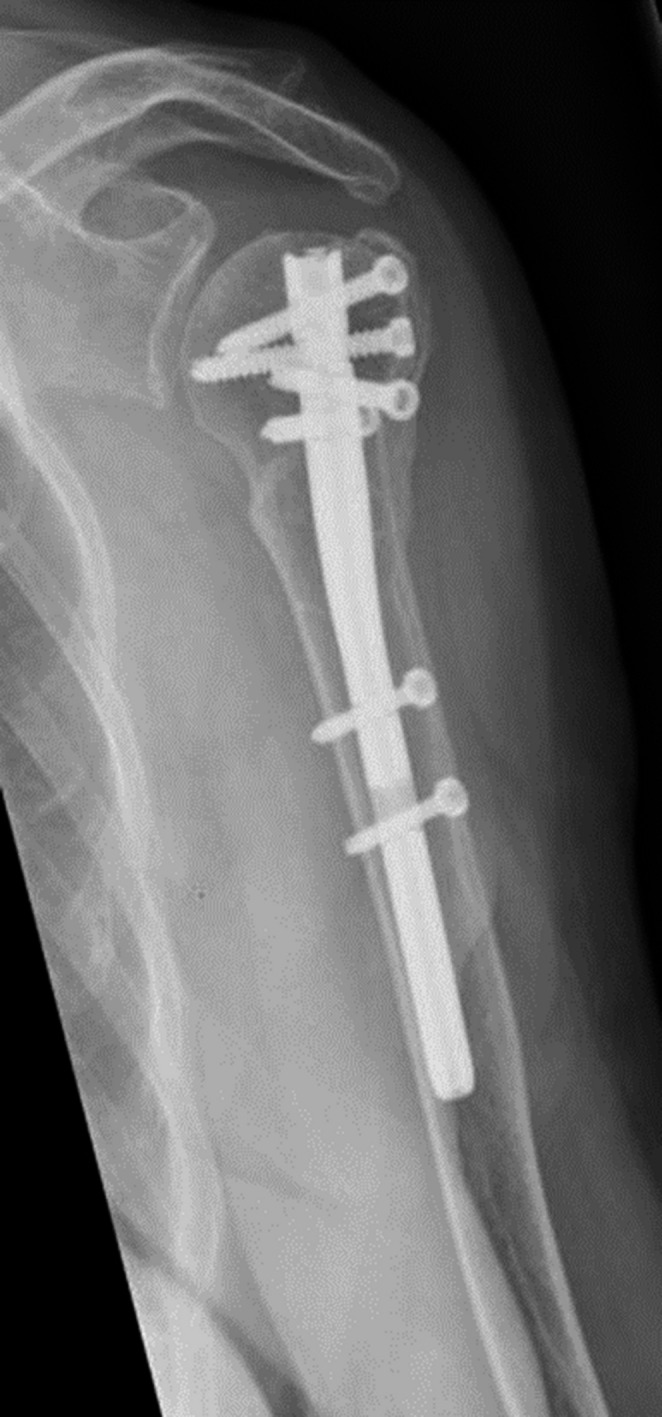
Healed fracture in AP-view

**Fig. 13:**
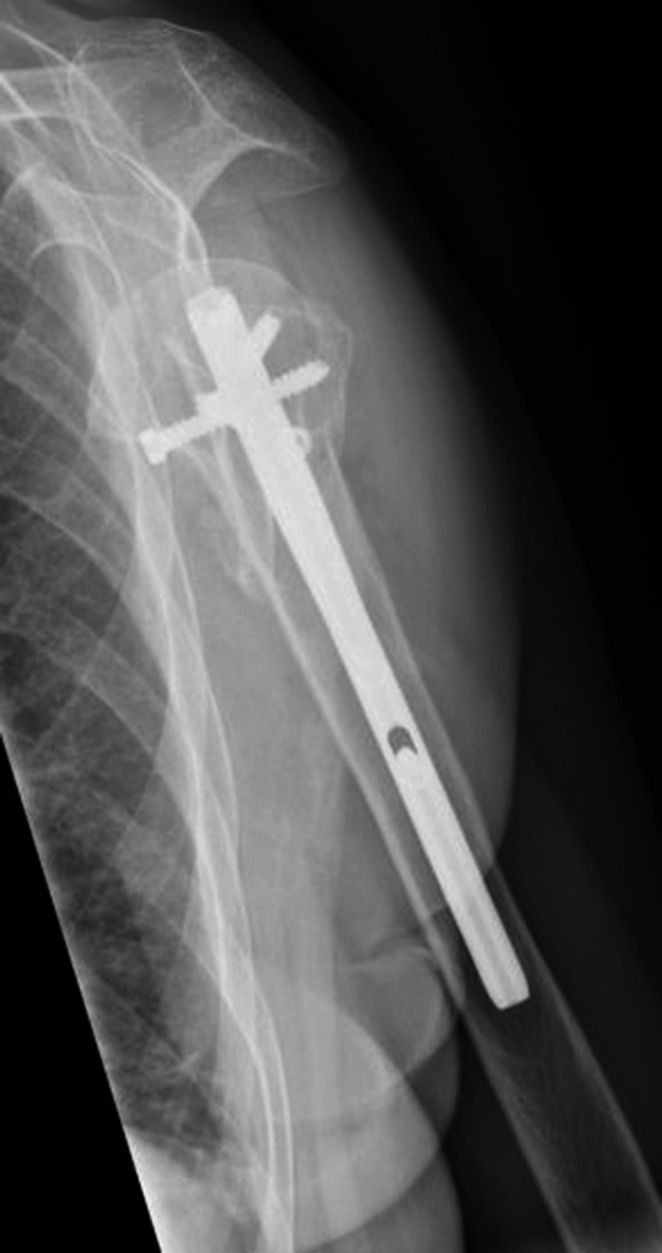
Healed fracture in lateral view

The postoperative course was uneventful. She was dismissed on the third postoperative day and follow up was continued in the outpatient clinic. Function recovered gradually under physiotherapy and regular X-rays showed healing of the fracture (Figs. 12 and 13). After a 2 year follow-up the fracture was completely healed and function was well restored with both an abduction and forward flexion till 160°. The Constant & Murley score was 64 points for both sides.

## Discussion

Healing and functional outcomes following intramedullary nailing are generally favorable and comparable across various studies (Table 1). Healing rates in most studies exceed 90% [2, 14]. Functional outcomes are similarly positive, with mean Constant-Murley (CM) scores ranging from 50 to 82 points [1, 15, 17]. However, most studies include a mix of younger and older patients, with results for elderly patients being significantly lower. Popescu et al. specifically examined outcomes in elderly patients within a cohort of 28 individuals, 64% of whom were over 70 years old. The functional outcomes for this group were notably worse compared to the younger cohort, with mean CM scores of 79.33 and 59, respectively [1]. Giannoudis et al. reported a mean CM score of 74.5, with patients older than 60 years exhibiting significantly lower scores [18]. Mathews et al. investigated the treatment of proximal humeral fractures in the geriatric population, treating 39 patients with a mean age of 81 years. The mean CM score in this cohort was 57 [19]. Sosef et al. reported a mean CM score of 62 [2]. The observed differences in CM scores may be explained by the varying functional demands of different age groups. In general, adults require approximately 120° of forward elevation, 45° of extension, 130° of abduction, 115° of cross-body adduction, 60° of external rotation, and 100° of internal rotation for normal shoulder function. However, several studies have shown that establishing full range of motion is not necessary for ADL (activities of daily living ) [20–22]. Moreover, in case of the elderly population multiple research have shown that shoulder range of motion will decrease with aging, without any decrease in functional ability, especially in patients with lower functional demands [23, 24]. Range of motion normally decreases during aging owing to changes in the musculoskeletal system, including loss of tissue elasticity and decreased muscle mass. This was supported by Fiebert et al., who suggested that joint capsules, ligaments, muscles and tendons shortened as part of normal ageing, causing decreased range of motion. For many elderly patients with proximal humeral fractures, regaining the ability to perform ADL and its social and emotional implications, which are best measured using PROMs, are often more important than regaining maximal range of motion and strength. If the authors look further with regards to the expected range of motion in the shoulder of the elderly there is some variability. The range of motion measurements reported by the American Academy of Orthopedic Surgeons are generally referred to as the main source of normative data [25–27]. However, these have been criticized since the AAOS failed to describe the population used to obtain them according to age, gender and occupation [25, 26]. In contrast, others have found that expected shoulder range of motion in elderly is, in fact, less than the AAOS norms [27–29]. The authors conclude that the minimal important range of motion depends on the functionality of the patient, were we are convinced that in the elderly 90° of abduction and/or anteflexion will provide most needs in daily activities (brushing teeth, combing hair). Improvement should continue for 12 to 18 months; with most people regaining a functional range of movement to perform day to day activities but one may not achieve full range of movement.

**Table 1:** List of publications

Author	Year of publication	Study type	Intervention	*n*	Age (y)	% Female	% Follow up	% Healing	% Complications
Rajasekhar C et al. [13]	2001	retrospective	Polarus Nail	30	71	53	80	96	16
Mittlmeier TW et al. [17]	2003	retrospective	Targon Nail	221	69	70	29	97	51
Agel J et al. [14]	2004	retrospective	Polarus Nail	20	48	40	100	65	30
Popescu D et al. [1]	2009	retrospective	T_2_ Proximal Humeral Nail	28	66,5	61	68	100	7
Sosef N et al. [2]	2010	retrospective	T_2_ Proximal Humeral Nail	33	78	25	70	95	38
Hatzidakis AM et al. [32]	2011	retrospective	T_2_ Proximal Humeral Nail	48	65	74	79	100	13
Zhu Y et al. [33]	2011	prospective randomised trial	PHN Synthes/Locking plate	26/25	54,8/50,9	64/69	100/100	100/100	4/31
Giannoudis PV et al. [18]	2012	retrospective	Polarus Nail	25	61	72	93	96	26
Fazal MA et al. [3]	2014	retrospective	Polarus Nail	46	63	59	100	96	33
Boudard G et al. [10]	2014	retrospective	Nail/Plate	32/35	64/49,6	63/41	94/94	?	43/62
Kloub M et al. [11]	2014	retrospective	Targon Nail	137	61	75	91	100	59
Liu QH et al. [15]	2014	retrospective	Trigen Nail	64	71	81	81	100	11
Plath JE et al. [31]	2019	prospective randomised trial	Locking Blade Nail/Philos plate	36/32	71/77	72/78	81	?	33/34
Wikeroy AKB et al. [30]	2025	prospective randomised trial	Multiloc Nail/Philos Plate	38/41	66/67	87/82	100/93	100/100	37/11

A recent prospective randomized controlled trial, comparing intramedullary nails with plating, showed no statistically significant differences in patient-reported outcomes or function two years after surgery for fixation of displaced 3- and 4-part part proximal humerus fractures [30]. However, Plath et al. found that in the elderly (mean age 76 years) and the majority of 3-part fractures, significant better DASH scores were noted 12 months postoperatively in the nail group compared to the patients treated with plate osteosynthesis. A significantly higher rate of secondary loss of reduction with concomitant screw penetration was seen in the PHILOS cohort [31].

Both for plates and intramedullary nails high rates of complications have been reported, like screw perforation of the head, avascular head necrosis (AVN) and secondary displacement.

According to Giannoudis et al., the AVN was 1.9% overall. Loosening of proximal locking screws varied from 3.7–15% [18]. Matthews reported implant-related complications in 15% of cases [19] Hatzidakis et al. reported 11% re-interventions [32]. Mittlemeier mentioned backing out of screws in 22.6% of cases with an AVN of 7.8% [17]. Sosef had implant-related complications in 12% [2]. Popescu mentioned implant-related complications in 17% [1].

In general complication ratio is comparable between plate and nail osteosynthsis [33, 34]. However some studies show that the intramedullary nail is superior to locking plate in reducing the total complication rate, intraoperative blood loss, operation time, postoperative fracture healing time and postoperative humeral head necrosis [31, 35]. The literature however still shows that compared with non-operative treatment any surgical treatment of proximal humeral fractures does not lead to better functional results and most likely results in more surgical interventions to cope with complications [36, 37].

## Conclusion

Based on the current literature we can conclude that the intramedullary nail is a valid technique in the treatment of proximal humeral fractures in the elderly. Results and complication rates are comparable with plate osteosynthesis. Recent literature however shows some indications that compared to the plate, a nail has a lower complication rate, less intraoperative blood loss, shorter operation and fracture healing time and less postoperative humeral head necrosis.

It is the surgeon however who has to decide which is the best treatment option based on the patient’s demands and fracture characteristics, taking his own skills and experience into consideration.

## Data Availability

No datasets were generated or analysed during the current study.
